# Novel Approaches for Overcoming Biological Barriers

**DOI:** 10.3390/pharmaceutics14091851

**Published:** 2022-09-02

**Authors:** Vibhuti Agrahari, Prashant Kumar

**Affiliations:** 1 Department of Pharmaceutical Sciences, University of Oklahoma Health Sciences Center, 1110 N. Stonewall Avenue, Oklahoma City, OK 73117, USA; 2 Vaccine Analytics and Formulation Center, Department of Pharmaceutical Chemistry, University of Kansas, Lawrence, KS 66047, USA

The human body poses a spectrum of biological mechanisms operating at different levels that are important for its normal functioning and development. Due to the complex nature and varying properties of the body’s biological barriers, the development of novel drug delivery systems to specific targets represents both a challenge as well as an opportunity. Extensive attempts have been made to overcome the barriers that prevent entry of therapeutic drugs and vaccines necessary for the treatment of several diseases, impaired conditions or use as prophylaxis. Novel technologies or approaches focusing on overcoming the specific barriers have been studied by investigators in this field to address specific needs. The delivery of a wide range of therapeutic molecules across a variety of barriers is now possible, as experimentally demonstrated by various in vivo and in vitro systems. This Special Issue on the novel approaches for overcoming biological barriers is a collection of efforts led by the several investigators and their groups to address the unmet need and development of the advanced drug delivery systems.

Among all the barriers in the human body, the blood–brain barrier (BBB) has always been challenging, despite several efforts made over the decades to breach it. The first article of this Special Issue by Hugon et al. corroborates the application of a [^18^F] 2-fluoro-2-deoxy-sorbitol ([^18^F]FDS) PET imaging technique as a translational and quantitative marker of BBB permeability to probe the impact of spatially controlled focused ultrasound (FUS) together with microbubbles on the integrity of the BBB in mice for the first time [1]. [^18^F] FDS PET imaging presents a sensitive, quantitative, and noninvasive marker of BBB permeability. Other advantages of [^18^F] FDS PET include its safety, low MW, low distribution across the intact BBB, and low diffusion from the sonicated volume to the non-sonicated brain with an intact BBB over time scales of minutes [1]. In another study, Taweel et al. demonstrated the direct delivery of intranasal zolmitriptan to rat brain using zolmitriptan-loaded bilosomes in a mucoadhesive in situ gel [2]. This technique involving an in-situ gelling system has high viscosity and therefore extends nasal mucociliary transit time by resisting mucociliary clearance. This system is a promising intranasal substitute with boosted therapeutic effect for treating patients suffering from migraines [2]. In the next article, Chen et al. summarized several clinical and preclinical investigations of monoterpenoid Perillyl alcohol (POH), a naturally available anti-cancer agent for overcoming biological barriers [3]. The application of POH for intranasal, nose-to-brain, intra-arterial BBB delivery and permeation-enhancing functions are noteworthy. The authors also described the use of POH in combination with other therapeutic agents for the creation of new entities with enhanced transport across biological barriers. Thus, POH underlies its ability to overcome the obstacles placed by different types of biological barriers and accordingly shape its multifaceted promise in the drug development for cancer therapy [3]. In a novel approach, Jafari et al. demonstrated proof-of-principle of a hybrid electropermanent magnets (EPM)-based device for temporarily opening the BBB using both an in vitro cell culture and an in vivo mice model [4]. This technique is useful for safe and effective drug transport across the BBB and can selectively target different parts of the brain by tailoring electrical waveforms [4]. Corti et al. compiled the development and application of a derivative of tumor necrosis factor-α (TNF) to target the blood–brain–tumor barrier (BBTB) [5]. A peptide–cytokine fusion (NGR-TNF) was prepared by combining TNF to Cys-Asn-Gly-Arg-Cys-Gly peptide (NGR), a ligand of aminopeptidase N (CD13)-positive tumor blood vessels. NGR-TNF demonstrated effective TNF delivery to tumor vessels by overcoming the biological barriers restricting drug penetration in cancer lesions [5]. In comparison to other TNF-related drugs, extremely low-doses of NGR-TNF or its derivativities were able to successfully overcome the BBTB. Choi et al. discussed the recent advances in the targeted delivery of exosomes to the brain [6]. Exosomes carry various membrane proteins (e.g., CD9, CD63, PTGFRN, and Lamp2b) and lipids (e.g., phosphatidylserine) that can serve as the targeting moieties. Thus, gaining attention for their potential for natural BBB crossing, broad surface-engineering capability, promising results for CNS delivery, and potential as next-generation therapeutics for treating CNS diseases. This comprehensive review also highlights receptor-mediated transcytosis (RMT) as one of the widely investigated methods to cross the BBB for drug delivery to the central nervous system (CNS). Drugs can hijack RMT by expressing specific molecules that bind to RMT, such as the transferrin receptor (TfR), low-density lipoprotein receptor (LDLR), and insulin receptor (INSR). Cell-penetrating peptides and components of neurotropic viruses have also demonstrated efficient drug delivery across the BBB [6]. The next article is focused on the Zika virus (ZIKV). It is a global concern because it invades the brains of adults and fetuses. The investigations by Todorovski et al. described that Placenta-Crossing Peptide-Porphyrin Conjugates (PPCs) exhibited BBB translocation capacity in a mouse model that could potentially fill the Zika virus (ZIKV) treatment gap [7]. This group recently evaluated the in vitro BBB and blood–placental barrier (BPB) crossing ability and anti-ZIKV activity of eight new PPCs. They identified PP-P1 as the most promising candidate, with elevated trans-BBB and -BPB scores and the highest antiviral potency and high serum stability, with a t_1/2_ > 22 h, which bodes well for in vivo application [7]. Thus, peptide–porphyrin conjugation is a promising strategy to tackle brain-resident viruses. Alshammari et al. developed a non-invasive therapy using Ruboxistaurin (RBX) nanoparticles incorporated into the polyamidoamine (PAMAM) dendrimer generation 5 for the treatment of diabetic retinopathy [8]. This nanoformulation possesses high drug loading capacity and is safe on the human retinal macroglial Müller cells (MIO-M1). Thus, the nanoformulation developed in this study holds promise to improve the therapeutic outcomes of anti-VEGF therapy and the bioavailability of RBX to prevent vision loss, overcome ocular barriers, and increase patient adherence [8]. Wang et al. provide insight into the drug–enhancers–Carbomer Hydrogel (CP) and drug–enhancers–skin interactions and the structural characteristics of enhancers to ground the drug-specific molecular mechanisms of enhancers and pharmaceutical hydrogel design [9]. A systematic approach was established to evaluate the enhanced release and retention of whitening agents from CP hydrogel in the presence of enhancers based on interactions between drugs, enhancers, and CP or skin. In conclusion, this study provides a strategy for the reasonable utilization of enhancers and formulation optimization in topical hydrogel whitening. [9]. Zafar et al. rationally designed the nanolipid-based formulation of diclofenac (DC) for the treatment of inflammation to be administered by the oral route [10]. The bilosomes (BC) nanoformulation showed a prolonged DC release with high permeation flux in the in vitro release and ex vivo permeation study. The pharmacokinetic and pharmacodynamics studies revealed the enhanced bioavailability and anti-inflammatory activity of BC-DC compared to pure DC and DC-Liposomes (LP) [10]. Therefore, the developed nanoformulation improves the therapeutic efficacy and overcomes complications of gastric irritation and ulcers. In another study, Chu et al. developed a novel propofol-mixed micelle as a clinical alternative for anesthetics [11]. This novel micellar formulation reduces injection-site pain and the risk of hyperlipidemia due to the low content of free propofol and low-lipid constituent. In addition, the developed formulation overcomes the biological barrier of the reticuloendothelial system and complications of the marketed propofol formulation [11]. The rat paw-lick study showed a significant reduction in pain compared to Diprivan. Thus, overcoming the major problem of the commercial formulation. Notably, the novel propofol formulation had a non-hemolytic reaction and exhibited a good safety profile, displayed similar anesthetic actions, absorption, and clearance effects after a single dose in comparison with the marketed formulation [11]. In a novel approach, Tyagi et al. developed a pramlintide–silica microparticle hydrogel depot which offers a significant advantage for the formulation (in near-native conditions) and delivery (degraded products do not destabilize protein structure) of biologicals [12]. The sustained delivery of Pramlintide from the silica depot was investigated in a rat model for two-months after subcutaneous administration [12]. In conclusion, the injectable, scalable, and biodegradable silica-based delivery system has great potential for therapeutic applications and can mitigate risk factors and compliance issues related to multiple dosing of different drugs. Nimma et al. findings revealed that a Telmisartan–(CARP-1) functional mimetic–Osimertinib (TLM_CFM-F_OSM) combination has a superior anti-cancer effect in the treatment of non-small-cell lung cancers (NSCLC) by affecting multiple resistant markers that regulate mitochondrial homeostasis, inflammation, oxidative stress, and apoptosis [13]. EGFR-tyrosine kinase inhibitors (TKIs) are the leading therapy for a substantial percentage of NSCLCs. This group studied OSM (which targets EGFR T790M mutation and inhibits activation of AMPK/Lamin-B2/MAPK and PI3K/AKT) in combination with CFM 4.17 NLPFs (CARP-1 signaling and EGFR activity is inhibited by interacting with EGFR’s ATP binding site) and TLM (disrupts tumor stromal barriers and leads to enhanced permeation of drugs) and proposed that it will provide superior anti-cancer effects in NSCLC and identify novel targets in tumor regression by using RNA sequence and quantitative proteomics. [13]. The comprehensive review by Plaunt et al. [14] highlighted the recent examples of how the barriers of pulmonary delivery can be overcome using formulation technologies or modifying the chemistry of the compound (drug).

Through decades of effort, scientists, chemists, biologists, toxicologists, engineers, etc., have developed solutions to overcome, and in some cases, even capitalize on, the numerous barriers of the human body as described above. Nonetheless, the relevance of these biological barriers differs by disease, and strategies to overcome barriers are specific to the target-site or treatment. Altogether, this Special Issue incorporates recent advances in overcoming these biological barriers by various research groups. This compilation will bring great opportunities to the scientific community engaged in the research and development of advanced drug delivery systems, and we expect that readers will find this issue an interesting addition to the existing literature.
